# Efficacy of Intraoperative Paracetamol and Nefopam Infusions in Addition to Transversus Abdominis Plane Block in Kidney Transplant Recipients

**DOI:** 10.3390/medicina61010065

**Published:** 2025-01-02

**Authors:** Jaesik Park, Sun Cheol Park, Min Suk Chae, Sang Hyun Hong, Jung-Woo Shim

**Affiliations:** 1 Department of Anesthesiology and Pain Medicine, Seoul St. Mary’s Hospital, College of Medicine, The Catholic University of Korea, Seoul 06591, Republic of Korea; alphanzx@gmail.com (J.P.);; 2 Department of Surgery, Division of Vascular and Transplant Surgery, Seoul St. Mary’s Hospital, College of Medicine, The Catholic University of Korea, Seoul 06591, Republic of Korea

**Keywords:** paracetamol, nefopam, kidney transplant, multimodal analgesia, transversus abdominis plane block

## Abstract

*Background and Objectives*: Kidney transplantation (KT) is an important treatment modality for renal failure. However, moderate-to-severe pain often occurs in KT recipients. Multimodal analgesia using combined analgesic measures has been recommended to enhance postoperative recovery. This retrospective study explored the additional analgesic efficacy of paracetamol and nefopam infusions in living-donor KT recipients who received a transversus abdominis plane (TAP) block. *Materials and Methods*: Consecutive living-donor KT recipients at our institute between January 2020 and March 2022 were divided into groups that received a TAP block with paracetamol and nefopam infusions (Group TA) or a TAP block without analgesics (Group T) during surgery. Following propensity-score (PS) matching, 103 patients were included in each group. Postoperative pain intensity assessed using the visual analog scale (VAS), opioid consumption via patient-controlled analgesia (PCA) devices over 24 h, and postoperative outcomes were compared between the two groups. *Results*: VAS pain intensity at rest was lower in group TA than in group T at 1 and 6 h after surgery [1 h: 29 (15–41) vs. 41 (29–51) mm, *p* < 0.001; 6 h: 32 (23–43) vs. 40 (32–54) mm, *p* < 0.001]. The VAS pain intensity during coughing was lower in group TA [1 h: 46 (30–58) vs. 59 (48–69) mm, *p* < 0.001; 6 h: 51 (40–63) vs. 60 (45–71) mm, *p* < 0.001]. Moreover, PCA consumptions during the first 6 h and between 6–24 h post-surgery was significantly lower in group TA. Other postoperative outcomes did not differ between the two groups. *Conclusions*: Multimodal analgesia with intraoperative paracetamol and nefopam infusions improved postoperative pain control in living-donor KT recipients who received a preoperative TAP block. Our findings demonstrate the efficacy of paracetamol and nefopam infusions in KT recipients.

## 1. Introduction

Chronic kidney disease is a complex disease which occurs in approximately 13% of the world’s population, causing progression to end-stage kidney disease [1]. Currently, end-stage kidney disease is not curable and its management requires renal replacement therapy. With the development of perioperative care strategies, kidney transplantation (KT) has become the best therapeutic option for patients with renal failure [2]. In particular, living-donor KT results in better graft outcomes and lower mortality rates than deceased donor KT [3].

During the perioperative period, enhanced pain control has been emphasized to facilitate the recovery and rehabilitation of surgical patients [4]. Poorly controlled pain may increase morbidity, functional impairment, cost of care, opioid use, and persistent pain lasting for months after surgery [5]. KT recipients often experience moderate-to-severe pain; however, optimal pain relief is especially challenging because of clinical difficulties in administering analgesic measures [6,7]. Impaired kidney grafting may result in variable responses to systemic analgesics, and previous dialysis may cause platelet dysfunction and residual heparin effects, interfering with the use of epidural analgesia.

The transversus abdominis plane (TAP) block has been widely employed [8]. The surgical approach for KT requires a curvilinear incision extending from the anterior superior iliac spine to the pubic symphysis. Therefore, transversus abdominis plane block may be an appropriate regional analgesic method for KT. However, both parietal and visceral pain should be managed to provide optimal pain relief after abdominal surgeries [9]. Moreover, visceral pain is not covered by the TAP block alone, despite its proven benefits in reducing postoperative parietal pain and opioid requirements [10,11].

Recently, a multimodal analgesic approach using non-opioids with regional interventions has been recommended in the enhanced recovery protocols [12]. The concept of multimodal analgesia, using two or more analgesic medications, is derived from their synergistic effects along the pain pathway [13]. In line with this shift toward a multimodal analgesic approach, several studies have explored the effects of intraoperative systemic analgesic medications combined with regional analgesic measures to improve postoperative pain control [14,15].

Among various non-opioids, paracetamol is often preferred because of its versatility and cost-effectiveness [13]. Nefopam has also been noted as an emerging element in multimodal analgesia owing to its opioid-sparing effect and unique mechanism of action as a centrally acting analgesic [16]. Their non-opioid and nonsteroidal properties and safety in patients with renal dysfunction make them suitable for inclusion in multimodal analgesic regimens for KT recipients [17,18].

Although the application of multimodal analgesic strategies or the analgesic role of TAP block has been previously studied in KT recipients [17,19], few reports have evaluated the additional effects of paracetamol and nefopam infusions in patients receiving TAP block. Therefore, we aimed to investigate the impact of intraoperative paracetamol and nefopam on pain management, opioid requirements, postoperative renal function, and other surgical outcomes, along with a TAP block through a retrospective analysis of living-donor KT recipients at our institute.

## 2. Materials and Methods

### 2.1. Ethical Considerations

This retrospective cohort study was conducted at a single center at Seoul St. Mary’s Hospital. The hospital’s institutional review board and ethics committee approved this study on 6 May 2022 (approval number: KC22RISI0289). The requirement for informed consent was waived owing to the study design.

### 2.2. Study Population

The medical records of consecutive patients who underwent living-donor KT for end-stage kidney disease between January 2020 and March 2022 at the Department of Vascular and Transplant Surgery of our institute were comprehensively reviewed.

Living-donor KT recipients aged ≥19 years were considered eligible for inclusion in this study. The exclusion criteria were refusal to receive TAP block, any analgesic use other than that included in our multimodal analgesic protocol, chronic liver disease, hemodynamic shock, postoperative psychosis, reoperation, and severe PONV requiring cessation of patient-controlled analgesia (PCA) infusions within 24 h after surgery.

### 2.3. Living-Donor Kidney Transplantation

After the patient arrived in the operating room, routine monitoring devices such as a noninvasive blood pressure cuff, three-lead electrocardiogram, pulse oximeter, and central venous pressure were placed. Intravenous (IV) propofol (1.5–2 mg/kg, Fresenius Kabi, Bad Homburg, Germany) and rocuronium (0.8–1 mg/kg, Merck Sharp and Dohme Corp., Kenilworth, NJ, USA) were injected to induce anesthesia. To maintain balanced general anesthesia, 3–6% of inhaled desflurane (Baxter, Deerfeld, IL, USA) with 0.01–0.2 µg/kg/min of IV remifentanil (Hanlim Pharm. Co., Seoul, Republic of Korea) was provided with a target hypnotic depth between 20 and 60 using a Bispectral Index instrument (Medtronic, Minneapolis, MN, USA).

In KT recipients, an inverted J-shaped curvilinear incision extending from the anterior superior iliac spine to the pubic symphysis was initially made and the pelvic fossa was exposed. For all donors, hand-assisted laparoscopic donor nephrectomy was performed, and the kidney graft was cooled on ice prior to implantation into the recipient [20]. After flushing with the preservation solution, the kidney graft was transplanted into the recipient. Following the end-to-side anastomoses between the recipient external iliac artery and vein and the graft renal artery and vein, ureteroneocystostomy was conducted with insertion of a double-J stent using the Lich-Grègoir technique. The vascular anastomosis and renal pedicle area were reassessed with careful hemostasis. Finally, the peritoneum, subcutaneous tissue, and skin were closed layer by layer after the Jackson–Pratt drain was placed.

During KT, the central venous pressure (CVP) was monitored using a central venous catheter (Arrow, Morrisville, NC, USA). To adjust the mean arterial pressure to ≥65 mmHg, dopamine (Reyon Pharm. Co., Seoul, Republic of Korea) was infused at a rate of 5–10 µg/kg/min. To increase urinary flow, 20–50 g of mannitol (Daihan Pharm. Co., Seoul, Republic of Korea) was administered for 10 min before reperfusion [21], and 20–40 mg of furosemide (Handok Pharm. Co., Seoul, Republic of Korea) was administered after reperfusion. However, routine monitoring of the arterial blood pressure or blood gas analysis using radial artery cannulation was not yet performed to prevent arterial injury. At the discretion of the attending anesthesiologist, balanced crystalloid fluids, including 0.9% normal saline (Daihan Pharm Co., Seoul, Republic of Korea) and Plasma Solution-A (CJ Healthcare, Seoul, Republic of Korea), were infused during surgery. Maintenance fluid doses were determined by considering the patient’s weight and anticipated tissue trauma [22]. Additional fluid boluses were infused to maintain a CVP of 10–15 mmHg or a hydration volume of 50–100 mL/kg, ensuring adequate graft perfusion by promoting sufficient renal blood flow and compensating for urinary volume drained after renal artery declamping [23]. At the end of the surgery, the neuromuscular blockade was reversed using sugammadex (4 mg/kg; Merck Sharp and Dohme Corp., Kenilworth, NJ, USA). Patients were extubated after confirmation of spontaneous eye opening and a train-of-four ratio of ≥90%.

For immunosuppression in KT recipients, the induction agents were interleukin-2 receptor antagonists and T lymphocyte-depleting rabbit-derived anti-thymocyte globulin, whereas the maintenance agents were calcineurin inhibitors, mycophenolate mofetil, and steroids. Graft rejection was treated using steroid pulse therapy and/or thymoglobulin rescue therapy.

### 2.4. Multimodal Analgesic Protocol and Pain Measurements

Established in December 2019, our multimodal analgesic protocol for living-donor KT recipients includes an abdominal wall block using a TAP block and preventive administration of non-opioid analgesic drugs.

An ultrasound-guided TAP block was performed by expert anesthesiologists on the surgical side after anesthesia induction. A linear ultrasound probe (Affiniti 70 Ultrasound System, Philips, Amsterdam, The Netherlands) was placed between the iliac crest and the lower costal margin in the mid-axillary line. After visualizing the three layers of the abdominal wall muscle, a sterile 21-G 8.5 mm block needle (Echoplex; Vygon Co., Paris, France) was carefully advanced in the medial-to-lateral direction using an in-plane technique. Subsequently, 20 mL of 0.375% ropivacaine (Mitsubishi Tanabe Pharm. Co., Osaka, Japan) was injected into the plane between the transverse abdominis and internal oblique muscles.

Our analgesic protocol included the administration of 1 g of paracetamol (Woosung Pharm. Co., Suwon, Gyeonggi-do, Republic of Korea) and 20 mg of nefopam (Myungmoon Pharm. Co., Seoul, Republic of Korea) infusions 15 min after reperfusion of the kidney graft. However, the retrospective chart review indicated that intraoperative paracetamol and nefopam infusions were administered in approximately 60% of living-donor KT recipients, while preoperative TAP blocks were performed in all cases.

After KT, all recipients received IV PCA (AutoMed 3200; Acemedical, Seoul, Republic of Korea). The regimen included a mixture of 1000 µg of fentanyl (Dai Han Pharma. Co., Seoul, Republic of Korea) and 0.3 mg of ramosetron (Boryung Pharma. Co., Seoul, Republic of Korea) diluted in normal saline to a total volume of 100 mL. The PCA was set to deliver a basal infusion rate of 1 mL/h with a 1 mL bolus and a lockout interval of 10 min. The patients were instructed to use a PCA bolus when a breakthrough pain with intensity ≥ 40 mm on the visual analog scale (VAS) occurred. In cases of severe PONV that did not respond to rescue antiemetics, PCA infusion was discontinued and 50 mg of tramadol (Yuhan Pharma. Co., Seoul, Republic of Korea) was infused according to the patient’s demands for rescue pain control.

The attending nurses measured the VAS pain intensity of all recipients at 1, 6, and 24 h after surgery (0–100 mm; 0 = no pain and 100 = worst possible pain) [24]. Static pain intensity was assessed at rest (VAS-R), whereas dynamic pain intensity was assessed during coughing (VAS-C). Cumulative PCA requirements were also recorded 6 and 24 h postoperatively.

Among the components of our multimodal analgesic protocol for living-donor KT recipients, the compliance rate for intraoperative paracetamol and nefopam infusions was approximately 60%, as previously mentioned. This allowed us to compare the analgesic efficacy between the TAP block with the analgesics group (Group TA) and TAP block without the analgesics group (Group T) by dividing patients into the two groups.

### 2.5. Clinical Variables

Demographic data such as age, sex, body mass index, and medical history were recorded. The surgical data included surgical duration, fluid balance (fluid infusion, blood transfusion, urine output, and estimated blood loss), and donor graft parameters (total ischemic time, graft site, and weight). Perioperative renal function was quantified based on the estimated glomerular filtration rate (eGFR), calculated using the Modification of Diet in Renal Disease formula: eGFR = 175 × standardized serum creatinine − 1.154 × age − 0.203 × 1.212 (if black) × 0.742 (if female). Postoperative data, such as laboratory findings; delayed graft function, defined as the need for dialysis within 1 week after surgery; any complications, including hemodynamic shock, psychosis, and PONV; and duration of hospital stay, were also checked.

The patients in Group TA were propensity-score (PS) matched to the patients in Group T based on sex, age, body mass index, native kidney primary diagnosis, eGFR, surgical duration, doses of fluid infusion, urine output, estimated blood loss, total ischemic time, graft site, and weight.

The primary outcome of this study was the VAS pain intensity at rest and during coughing at 1, 6, and 24 h after surgery. Secondary outcomes were postoperative PCA consumption expressed in morphine equivalents, renal function, PONV, and surgical complications.

### 2.6. Statistical Analyses

The normality of continuous data was assessed using the Shapiro–Wilk test. Descriptive statistics were reported as frequencies (with percentages) for categorical variables and means (with standard deviations) or medians (with interquartile ranges and 10–90 percentile range) for continuous variables. PS matching analyses were performed to reduce the effects of potential confounding factors on intergroup differences. A 1:1 nearest-neighbor, PS matching was conducted without replacement. Categorical variables were compared using the chi-squared test. Student’s *t*-test or the Mann–Whitney U test was performed to compare continuous variables according to the normality of the distribution. Two-tailed tests were performed for all statistical analyses and *p* < 0.05 was considered to be statistically significant. All analyses were performed using SPSS Statistics software (version 26.0; IBM Corp., Armonk, NY, USA).

## 3. Results

### 3.1. Enrollment Information of the Study

The study population included 313 consecutive living-donor KT recipients at Seoul St. Mary’s Hospital between January 2020 and March 2022. A review of their perioperative records showed that the following patients met the exclusion criteria: two patients who refused to receive a TAP block; three patients with chronic liver disease; seven patients with hemodynamic shock; seven patients with postoperative psychosis; 14 patients with severe PONV; and five patients who underwent re-operations. On the other hand, none of the study patients were administered any analgesic drug other than those included in our multimodal analgesic protocol.

**Figure 1:**
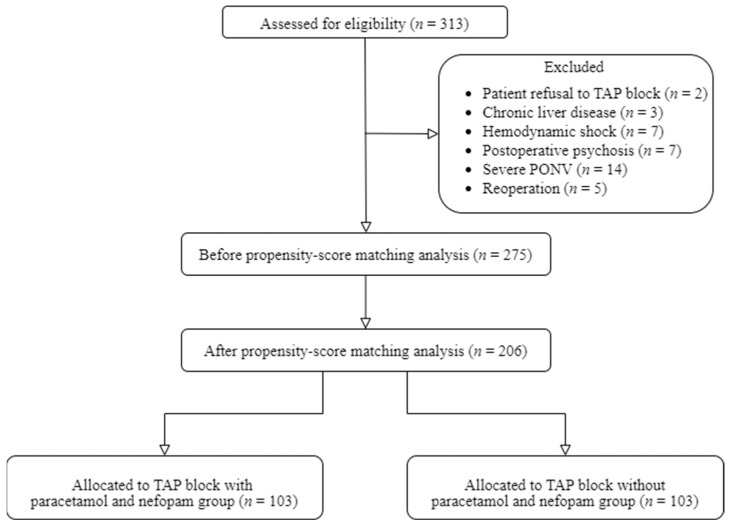
Flow chart of the study. TAP, transversus abdominis plane; PONV, postoperative nausea/vomiting.

Of the remaining 275 patients, 158 received intraoperative paracetamol and nefopam infusions (Group TA), while the other 117 did not (Group T). After applying PS matching analysis, 206 patients (103:103) patients were successfully matched with standardized mean differences of less than 0.15 for all variables (Figure 1).

### 3.2. Comparison of Perioperative Factors Between the Groups of Patients Who Received a TAP Block With or Without Paracetamol and Nefopam Infusions Before and After PS Matching

Before PS matching, significant differences were observed between the two groups in terms of surgical duration and fluid infusion. However, among PS-matched patients based on the aforementioned factors, there were no significant differences in perioperative factors between the two groups (Table 1).

### 3.3. Comparison of Postoperative Analgesic Profiles Between the Groups of Patients Who Received a TAP Block With or Without Paracetamol and Nefopam Infusions Among PS-Matched Patients

Table 2 shows that the VAS-R score was significantly lower in group TA than in group T at 1 and 6 h after surgery [1 h: 29 (15–41) vs. 41 (29–51) mm, *p* < 0.001; 6 h: 32 (23–43) vs. 40 (32–54) mm, *p* < 0.001]. The VAS-C score was significantly lower in group TA than in group T at 1 h and 6 h after surgery [1 h: 46 (30–58) vs. 59 (48–69) mm, *p* < 0.001; 6 h: 51 (40–63) vs. 60 (45–71) mm, *p* < 0.001]. PCA consumption during the first 6 h and between 6 and 24 h post-surgery was significantly lower in group TA compared to group T, [0–6 h; 13.5 (9.4–19.5) vs. 16.4 (11.4–23.5) mg, *p* = 0.005, 6–24 h; 30.6 (21.1–45.2) vs. 35.3 (23.4–52.7) mg, *p* = 0.048].

### 3.4. Comparison of Postoperative Outcomes Between the Groups of Patients Who Received a TAP Block With or Without Paracetamol and Nefopam Infusions Among Propensity-Score Matched Patients

As shown in Table 3, postoperative outcomes—such as eGFR, urine output, alanine aminotransferase level on the first postoperative day, incidences of PONV within 24 h, occurrences of delayed graft function, and duration of postoperative hospital stay—did not differ between the two groups. None of the patients experienced TAP block-related complications (nerve injury from the needle, hematoma, or local infection).

## 4. Discussion

The results of this study indicate that intraoperative paracetamol and nefopam infusions as part of multimodal analgesia improve acute postoperative analgesia in living-donor KT recipients who received a preoperative TAP block. Paracetamol and nefopam infusions reduced early postoperative pain, as measured by the VAS-R and VAS-C at 1 and 6 h. PCA consumption during 6–24 h was also reduced by intraoperative paracetamol and nefopam infusions as well as by PCA consumption until 6 h after surgery. However, there were no differences in postoperative renal function, PONV, or other surgical outcomes.

TAP blocks have been widely used for pain control in various abdominal surgeries, improving analgesia and patient satisfaction with recovery [8,25]. Furthermore, the development of an ultrasonography-guided approach has made it accessible to clinicians. By spreading local anesthetics along the abdominal fascial plane, the TAP block induces an analgesic action on incisional pain. However, sufficient analgesia may not be warranted when a TAP block is the sole analgesic measure because of the complex characteristics of pain after surgery [26]. Parietal pain is derived from well-localized skin, whereas visceral pain arises from the viscera, muscles, and bones with a diffuse and dull nature [9]. Although the clinical use of the TAP block is favored, previous studies by Kuruba et al. and Freir et al. questioned its analgesic efficacy in KT recipients [27,28]. Mukhtar et al. recommended that TAP blocks be administered as part of a balanced analgesic approach for KT recipients, rather than as a single method [29].

Multimodal analgesia refers to the combined use of different analgesic medications or interventions with distinctive mechanisms of action to maximize their beneficial effects with minimal side effects owing to synergism [13]. Among the various analgesic agents and routes of administration, acetaminophen is widely used in the commercially available IV form (paracetamol). Its centrally acting analgesic mechanism involves the inhibition of N-methyl-D-aspartate receptors and the cyclooxygenase-2 pathway [30]. Paracetamol has advantages over nonsteroidal anti-inflammatory agents in patients with end-stage kidney disease. However, paracetamol should be used with other analgesic agents to control moderate-to-severe pain [31].

In contrast, nefopam is known for its analgesic role as an adjunct to multimodal analgesia, for example, in combination with paracetamol [32,33]. Nefopam is a benzoxazocine analgesic drug that acts by inhibiting serotonin, norepinephrine, and dopamine reuptake and decreasing the activation of postsynaptic glutamatergic receptors, including N-methyl-D-aspartate receptors [34]. Because nefopam is a non-opioid, nonsteroidal, and non-sedating medication, it is not associated with side effects such as respiratory depression, sedation, and gastrointestinal or liver damage associated with opioid or steroidal medications [32]. Because of these properties, nefopam has been used clinically for postoperative pain management [35]. In a previous study, 20 mg of nefopam administered during surgery decreased acute pain and opioid use after laparoscopic gastrectomy [16]. In a meta-analysis by Zhao et al. [36], the efficacy and safety of nefopam during laparoscopic cholecystectomy were demonstrated.

Furthermore, Kim et al. demonstrated that nefopam (160 mg in 200 mL at a rate of 4 mL/h for 48 h after the graft reperfusion) reduced postoperative fentanyl requirements by 20% in KT recipients [18]. Consistent with this finding, group TA in our study showed a 20% reduction in fentanyl-PCA consumption until 6 h after surgery compared with group T. However, our study differed from that of Kim et al. in that the TAP block was performed in all patients, and 20 mg of nefopam was infused with 1 g of paracetamol for a short time during surgery [18]. The novelty of our findings lies in the focus on the intraoperative use of paracetamol and nefopam in living-donor KT recipients within a multimodal analgesic protocol including a preoperative TAP block. To the best of our knowledge, this topic has not yet been explored in the literature.

Paracetamol and nefopam were administered preventively in our study. Preemptive analgesia refers to providing analgesia preoperatively, not after surgical incision, to prevent central sensitization [37]. Preventive analgesia encompasses all perioperative measures used to decrease postoperative pain and analgesic requirements. Although it has been emphasized that analgesia should be preemptively initiated before incision or surgery, there is still debate regarding the ideal timing for perioperative analgesia [38]. Vadivelu et al. reported that the effective duration of a perioperative analgesic regimen was more important than the preoperative timing of a single analgesic measure [37]. Considering that the analgesic durations of paracetamol and nefopam were less than 6 h, the timing of their administration in our study after reperfusion of the kidney graft seems to be appropriate for optimal analgesia. The postoperative use of paracetamol and nefopam with an around-the-clock regimen may extend the duration of effective pain control [13].

Our study has several limitations. First, the nature of this single-center retrospective study inherently limits the establishment of causality and controls for all hidden biases. Despite this limitation, confounding factors related to patient and surgical characteristics were adjusted between the groups by applying PS matching analysis. Second, the degree of dermatomal spread of the TAP blocks was not confirmed, although all blocks were performed under ultrasound guidance. Third, the effect of the pain component (i.e., parietal vs. visceral pain) on analgesia after KT was not assessed. All patients were questioned about their overall pain intensity after surgery. Fourth, the optimal timing of analgesic administration (i.e., preemptive vs. preventive administration) was not evaluated because paracetamol and nefopam infusions were administered after graft reperfusion according to the analgesic protocol at our institute. Fifth, the analgesic effects of paracetamol and nefopam infusions in combination with other regional interventions, such as quadratus lumborum block, were not examined, as only TAP block was performed. Finally, this study did not evaluate which contributed more to enhanced postoperative pain control, paracetamol or nefopam infusions. Because coadministration of paracetamol and nefopam was performed in group TA, we assessed the analgesic efficacy of their coadministration in KT recipients who received a TAP block.

Despite these limitations, our study offers valuable insights into the additional effects of intraoperative paracetamol and nefopam infusions in KT recipients. Their efficacy should be validated in other types of abdominal surgeries requiring both parietal and visceral pain controls to generalize our findings. Moreover, further prospective studies examining their effects in combination with other analgesic approaches should be conducted to establish the ideal perioperative analgesic regimen.

## 5. Conclusions

The administration of intraoperative paracetamol and nefopam infusions led to additional analgesic benefit in living-donor KT recipients who had received a TAP block. When used as part of multimodal analgesia, intraoperative paracetamol and nefopam infusions reduced early postoperative pain scores as well as PCA consumptions, without increasing adverse effects.

## Figures and Tables

**Table 1:** Comparison of perioperative factors between the groups of patients who received TAP block with or without paracetamol and nefopam infusions before and after PS matching.

	Before PS Matching				After PS Matching			
Characteristics	TAP Block with Paracetamol and Nefopam (*n* = 158)	TAP Block Without Paracetamol and Nefopam (*n* = 117)	*p*-Value	SMD	TAP Block with Paracetamol and Nefopam (*n* = 103)	TAP Block Without Paracetamol and Nefopam (*n* = 103)	*p*-Value	SMD
Preoperative factors								
Age (years)	51 (41–58) [32–62]	51 (40–59) [30–64]	0.675	0.045	51 (44–58) [32–61]	50 (39–59) [30–65]	0.590	−0.088
Male sex	105 (66.5%)	82 (70.1%)	0.523	0.079	69 (67%)	71 (68.9%)	0.765	0.042
BMI	22.8 (20.2–25.1) [17.6–27.6]	23.3 (20.7–26.2) [19.4–29.4]	0.111	0.254	23.6 (21.4–26.3) [17.9–28.5]	22.8 (20.4–26.1) [19.1–29.4]	0.423	−0.044
Primary renal disease			0.595				0.999	
HTN	18 (11.4%)	11 (9.4%)		−0.068	10 (9.7%)	11 (10.7%)		0.031
DM	43 (27.2%)	34 (29.1%)		0.040	26 (25.2%)	28 (27.2%)		0.043
IgAN	31 (19.6%)	20 (17.1%)		−0.067	20 (19.4%)	18 (17.5%)		−0.051
Chronic GN	11 (7.0%)	11 (9.4%)		0.083	9 (8.7%)	10 (9.7%)		0.033
FSGS	4 (2.5%)	6 (5.1%)		0.117	4 (3.9%)	4 (3.9%)		<0.001
PKD	6 (3.8%)	1 (0.9%)		−0.318	1 (1.0%)	1 (1.0%)		<0.001
Others	45 (28.5%)	34 (29.1%)		0.013	33 (32.0%)	31 (30.1%)		−0.042
eGFR (mL/min/1.73 m^2^)	7.7 (5.7–9.8) [4.7–12.5]	7.4 (5.6–8.9) [4.3–10.7]	0.252	−0.314	7.2 (5.4–9.1) [4.6–12.6]	7.7 (6.0–9.0) [4.3–11.3]	0.626	0.025
Intraoperative factors								
Surgical duration (min)	215 (190–245) [170–265]	230 (200–263) [180–280]	0.015	0.272	220 (195–250) [180–282]	225 (195–255) [177–273]	0.773	0.016
Fluid infusion (mL)	2200 (1950–2600) [1650–2760]	2300 (2030–2680) [1690–3100]	0.041	0.270	2300 (2100–2600) [1820–2860]	2300 (2000–2650) [1670–3100]	0.841	0.046
Urine output (mL)	450 (269–520) [146–800]	400 (300–565) [200–850]	0.982	<0.001	420 (300–500) [150–800]	400 (300–580) [200–850]	0.960	<0.001
Estimated blood loss (mL)	175 (100–200) [100–300]	200 (100–200) [50–400]	0.085	0.176	200 (100–200) [100–300]	200 (100–200) [70–400]	0.434	0.137
Total ischemic time (min)	54 (45–67) [40–82]	54 (45–66) [40–76]	0.710	−0.078	54 (45–64) [41–80]	53 (44–66) [40–76]	0.594	−0.034
Graft site (left)	70 (44.3%)	54 (46.2%)	0.760	0.037	44 (42.7%)	48 (46.6%)	0.575	0.078
Graft weight (g)	175 (152–204) [138–236]	176 (148–209) [136–242]	0.850	0.003	168 (150–204) [139–244]	176 (150–216) [140–244]	0.443	0.099

Values are presented as medians (interquartile ranges) [10–90 percentile range] or number (proportion). PS, propensity score; TAP, transverse abdominis plane; SMD, standardized mean difference; BMI, body mass index; HTN, hypertension; DM, diabetes mellitus; IgAN, immunoglobulin A nephropathy; GN, glomerulonephritis; FSGS, focal segmental glomerulosclerosis; PKD, polycystic kidney disease; eGFR, estimated glomerular filtration rate.

**Table 2:** Comparison of postoperative analgesic profiles between the groups of patients who received TAP block with or without paracetamol and nefopam infusions among propensity-score matched patients.

	TAP Block with Paracetamol and Nefopam (*n* = 103)	TAP Block Without Paracetamol and Nefopam (*n* = 103)	*p*-Value
VAS pain score at rest (mm)			
At 1 h	29 (15–41) [5–54]	41 (29–51) [17–60]	<0.001
At 6 h	32 (23–43) [15–54]	40 (32–54) [20–64]	<0.001
At 24 h	17 (11–25) [6–36]	22 (13–29) [7–34]	0.071
VAS pain score during cough (mm)			
At 1 h	46 (30–58) [21–72]	59 (48–69) [33–78]	<0.001
At 6 h	51 (40–63) [24–73]	60 (45–71) [35–81]	0.008
At 24 h	35 (27–44) [20–54]	40 (27–50) [22–61]	0.089
PCA consumptions (morphine equivalents, mg)			
0–6 h	13.5 (9.4–19.0) [7.5–28.5]	16.4 (11.4–23.5) [9.9–26.9]	0.005
6–24 h	30.6 (21.1–45.2) [18.4–60.6]	35.3 (23.4–52.7) [18.7–66.8]	0.048

Values are presented as medians (interquartile ranges) [10–90 percentile range]. TAP, transversus abdominis plane; VAS, visual analog scale; PCA, patient-controlled analgesia.

**Table 3:** Comparison of postoperative outcomes between the groups of patients who received a TAP block with or without paracetamol and nefopam infusions among propensity-score matched patients.

	TAP Block with Paracetamol and Nefopam (*n* = 103)	TAP Block Without Paracetamol and Nefopam (*n* = 103)	*p*-Value
eGFR on POD 1 (mL/min/1.73 m^2^)	25.2 (17.7–40.3) [11.8–51.0]	25.1 (16.5–32.3) [11.1–43.3]	0.311
Urine output on POD 1 (L)	9.6 (8.3–11.2) [7.4–14.5]	9.2 (8.1–11.5) [7.1–13.6]	0.477
ALT (U/L) on POD 1	16 (12–27) [8–58]	15 (10–22) [6–41]	0.102
PONV until 24 h after surgery	12 (11.7%)	11 (10.7%)	0.825
Delayed graft function	3 (2.9%)	4 (3.9%)	0.701
Postoperative hospital stay (days)	13 (12–14) [12–17]	13 (12–14) [12–16]	0.783

Values are presented as medians (interquartile ranges) [10–90 percentile range] or numbers (proportions). TAP, transversus abdominis plane; eGFR, estimated glomerular filtration rate; POD, postoperative day; ALT, alanine aminotransferase; PONV, postoperative nausea and vomiting.

## Data Availability

The datasets generated and/or analyzed during this study are not publicly available because of ethical restrictions. Only de-identified datasets are available from the corresponding author upon reasonable request.
